# Combinatory Microarray and SuperSAGE Analyses Identify Pairing-Dependently Transcribed Genes in *Schistosoma mansoni* Males, Including Follistatin

**DOI:** 10.1371/journal.pntd.0002532

**Published:** 2013-11-07

**Authors:** Silke Leutner, Katia C. Oliveira, Björn Rotter, Svenja Beckmann, Christin Buro, Steffen Hahnel, Joao P. Kitajima, Sergio Verjovski-Almeida, Peter Winter, Christoph G. Grevelding

**Affiliations:** 1 Institute of Parasitology, Justus-Liebig-University Giessen, Giessen, Germany; 2 Departamento de Bioquímica, Instituto de Química, Universidade de São Paulo, São Paulo, Brasil; 3 GenXPro GmbH, Frankfurt Main, Germany; 4 Hospital Israelita Albert Einstein, São Paulo, Brasil; University of Queensland, Australia

## Abstract

**Background:**

Schistosomiasis is a disease of world-wide importance and is caused by parasitic flatworms of the genus *Schistosoma*. These parasites exhibit a unique reproduction biology as the female's sexual maturation depends on a constant pairing-contact to the male. Pairing leads to gonad differentiation in the female, and even gene expression of some gonad-associated genes is controlled by pairing. In contrast, no morphological changes have been observed in males, although first data indicated an effect of pairing also on gene transcription in males.

**Methodology/Principal Findings:**

To investigate the influence of pairing on males, we performed a combinatory approach applying SuperSAGE and microarray hybridization, generating the most comprehensive data-set on differential transcription available to date. Of 6,326 sense transcripts detected by both analyses, 29 were significantly differentially transcribed. Besides mutual confirmation, the two methods complemented each other as shown by data comparison and real-time PCR, which revealed a number of genes with consistent regulation across all methods. One of the candidate genes, follistatin of *S. mansoni* (SmFst) was characterized in more detail by *in situ* hybridization and yeast two-hybrid (Y2H) interaction analyses with potential binding partners.

**Conclusions/Significance:**

Beyond confirming previously hypothesized differences in metabolic processes between pairing-experienced (EM) and pairing-unexperienced males (UM), our data indicate that neuronal processes are involved in male-female interaction but also TGFβ-signaling. One candidate revealing significant down-regulation in EM was the TGFβ-pathway controlling molecule follistatin (SmFst). First functional analyses demonstrated SmFst interaction with the *S. mansoni* TGFβ-receptor agonists inhibin/activin (SmInAct) and bone morphogenic protein (SmBMP), and all molecules colocalized in the testes. This indicates a yet unknown role of the TGFβ-pathway for schistosome biology leading to male competence and a possible influence of pairing on the male gonad.

## Author Summary

Schistosomiasis is an important infectious disease caused by worm parasites of the genus *Schistosoma* and directly affects more than 240 million people in 78 tropical and sub-tropical countries but also animals. Pathogenesis is triggered by eggs that are produced by paired females and get trapped in liver and gut causing severe inflammation. While studies have concentrated on the reproductive biology of schistosome females in the past, not much is known about males even though they are indispensable for female sexual development and egg production. Therefore, we studied pairing-dependent processes in *S. mansoni* males using two independent transcriptomics approaches providing a congruent and most comprehensive data-set on genes being differentially transcribed between pairing-experienced, competent males and pairing-unexperienced, naive males. Besides confirming former studies concerning changes in metabolic processes, our results give new insights into processes leading to male competence indicating among others a potential role of neurotransmitters and TGFβ signal-transduction processes. We especially highlight the follistatin gene SmFst, which codes for an inhibitor of the TGFβ-pathway. SmFst transcription was localized in the testes and found to be down-regulated in pairing-experienced males. This indicates a yet unknown function of pairing on the male gonad and a further role of TGFβ-signaling for schistosome biology.

## Introduction

*Schistosoma mansoni* is a species of parasitic flatworms causing schistosomiasis, an infectious disease of worldwide importance for man and animals. Besides vertebrates as final hosts, the parasites' life cycle includes a snail intermediate host, and both are infected by aquatic larval stages. Schistosomiasis occurs in 78, mainly tropical and sub-tropical countries with about 600 million people at risk, of which 243 million required regular treatment in 2011. Thus it is one of the most prevalent parasitemias in the world, second only to malaria [1]–[4]. Pathology is induced by eggs deposited in the bloodstream by paired females, each producing up to 300 eggs per day [5]. A necessity for egg production is the completion and maintenance of the full development of female gonads. For this, the female depends on a constant pairing-contact to a male partner, an exceptional phenomenon in nature. Thus male worms have a key-role in the reproduction biology of schistosomes. Besides causing morphologic alterations comprising a significant increase of the body size of a paired female, which originates from mitogenic processes and differentiation of the reproductive organs ovary and vitellarium, the male even controls gene expression in its partner [6], [7]. While these effects on females have been a strong focus for research throughout the last decades [8]–[11], only few studies concentrated on pairing-dependent processes in the male. Authors of early studies presumed that different factors are transmitted from male to female controlling female body length as well as sexual maturation [12], [13]. Stimulation of the latter was reported to act locally [14], [15], also a tactile impulse was proposed, while sperm or seminal fluid were excluded [12], [14]. Vague evidence was found for a male-secreted hormone or protein to act on the female, however, none of these leads resulted in the identification of a concrete male stimulus [16]–[20]. Since glucose and other substances like cholesterol were shown to be transferred from the male to the female, also the supply of nutrients by the male was suggested to be the basis for female development [21]–[24].

So far only one glycoprotein (GCP = gynecophoral canal protein) was identified [25] and even described to be essential for pairing in *S. japonicum* [26]. Localization to the male gynecophoral canal as well as on the female surface further indicated the putative importance of GCP for the male-female interaction [27]. Although its function has not been clearly identified yet, evidence was obtained for the regulation of GCP by a TGFβ-dependent pathway in *S. mansoni* [28]. Other studies indicated that pairing may have an effect on the male as well, by influencing its capacity to stimulate mitosis in the female [29]. This led to the hypothesis that males have to reach a kind of competence before being able to induce developmental processes in the female.

Along with the progress of the genome [30]–[33] and large-scale transcriptome sequencing [34], [35] projects, additional analysis methods became available, which were used among others to compare pairing-experienced (EM) and pairing-unexperienced (UM) males. While the majority of these studies applied microarrays, several groups used serial analysis of gene expression (SAGE) alternatively. One of these studies [36] compared paired adult males and females and their pairing-unexperienced counterparts. For EM and UM the authors found differential regulation for transcripts contributing to developmental processes, metabolism and the redox-system. Already before the genome project was finished, an early microarray-based study compared EM and UM identifying 30 highly expressed genes to be exclusively transcribed in EM and 66 in UM [37]. Their identities indicated RNA metabolic processes to be differentially regulated between EM and UM, which was supported by a subsequent study [38].

Addressing the still unsolved question of male competence, here we investigated the influence of pairing on gene transcription in males. To this end we used two well established transcriptome analysis methods, SuperSAGE and microarray. The combination of both methods aimed at the production of corresponding data sets confirming, but also complementing each other to generate a comprehensive set of differentially transcribed genes in EM and UM that provides new insights into the male-female interaction. Among the most interesting genes identified here was a *S. mansoni* follistatin homolog (SmFst), a potential inhibitor of TGFβ pathways [39], [40]. Besides its pairing-dependent transcriptional regulation in males, our first functional analyses demonstrated not only gonad-preferential transcription of SmFst, but also its potential to interact with the TGFβ-receptor agonists SmInAct and SmBMP, which colocalized in the gonads. Thus, first evidence was obtained that TGFβ signaling plays an additional role for schistosome biology being one of probably several elements guiding male competence.

## Materials and Methods

### Parasite stock

The parasite life cycle was maintained using a Liberian isolate of *Schistosoma mansoni* [41], *Biomphalaria glabrata* as intermediate snail host, and Syrian hamsters (*Mesocricetus auratus*) as final host. To produce EM/EF (pairing-experienced males/females) or UM/UF (pairing-unexperienced males/females) snails were infected with either several miracidia (poly-miracidial infection), or only one miracidium (mono-miracidial infection). Poly-miracidial snail infections led to populations of male and female cercariae, which were used for bisex hamster infections resulting in EM and EF. Mono-miracidial snail infections led to unisexual populations of cercariae, which upon final-host infection developed into UM or UF. After 42 days (EM) or 67 days (UM) post infection adult worms were obtained by hepatoportal perfusion. This difference is due to our experience with experimental hamster infections that revealed a positive effect on perfusion efficiency and quality of unisexual worms, when the infection period is elongated to 67 days. This elongation had no influence on further experimental procedures, which concentrated on the comparison of the pairing status of male worms. For EM enrichment, paired males from bisex hamster infections were carefully separated from their partners by feather-weight tweezers, immediately frozen, and stored at −80°C until further use.

### Ethics statement

All experiments with hamsters have been done in accordance with the European Convention for the Protection of Vertebrate Animals used for Experimental and other Scientific Purposes (ETS No 123; revised Appendix A) and have been approved by the Regional Council (Regierungspraesidium) Giessen (V54-19 c 20/15 c GI 18/10).

### Isolation of RNA, cDNA-synthesis and standard PCRs

Total RNA from adult worms was extracted using TriFast (PeqLab) following the manufacturer's instructions. Subsequently extracted RNAs were quality-checked on denaturing formaldehyde gels.

Following total RNA extraction, cDNA synthesis was performed with the Quantitect Reverse Transcription Kit (Qiagen) following the manufacturer's protocol with 1 µg total RNA from EM or UM as template. Standard PCR reactions were performed in a final volume of 25 µl using primer end concentrations of 800 nM, an annealing temperature of 60°C, elongation at 72°C, and FirePol-Taq (Solis biodyne).

### Microarray-analyses

Following hamster perfusion, 50 EM or UM were collected, incubated over-night in RNAlater (Ambion) and stored at −80°C. For total-RNA isolation approximately 25 worms from each batch were washed twice in 500 µl H_2_O_DEPC_, followed by addition of 1 ml TRIzol reagent (Invitrogen). Subsequently, the worms were homogenized mechanically and incubated for 5 min at room temperature (RT) before 200 µl chloroform was added, mixed for 15 s and incubated for 2–3 min. Following centrifugation for 15 min at 12,000 g and 4°C the upper phase was transferred to a new tube and mixed with 500 µl Isopropanol. After incubation for 10 min at RT the RNA was centrifuged for 10 min at 12,000 g and 4°C. The pellet was washed with 1 ml ethanol (75%) and centrifugation repeated for 5 min at 4°C and 7,500 g. The supernatant was discarded, the pellet dried and resuspended in 25 µl H_2_O_DEPC_. Before determination of the concentration on a spectrophotometer (Nanodrop) the RNA was shortly heated to 65°C. RNA purification was done following the animal tissue protocol (Qiagen RNeasy Mini kit) with the following modifications: samples were supplemented to a volume of 100 µl, and 350 µl RLT buffer and 250 µl ethanol (70%) were added before the suspension was transferred onto a column (Qiagen RNeasy Mini kit). RNA was eluted with 30 µl H_2_O_DEPC_, and the flow-through put on the column for a second elution step. The concentration of RNA was determined again (see above), and its quality checked on a Bioanalyzer (Agilent 2100 Bioanalyzer, Agilent Technologies).

RNA was reverse transcribed, *in vitro* amplified, labeled with Cy3 or Cy5, and hybridized according to the Agilent technology protocol for “two color microarray based gene expression analysis”. The samples were hybridized on a 4×44 k oligoarray containing 60 mer oligonucleotides that was custom-designed by us [42], and manufactured by Agilent Technologies; the platform probe sequences are available on Gene Expression Omnibus (GEO) under the accession number GPL8606. This platform was recently re-annotated [43] according to the first draft of the genome project [30]. Three independent biological replicas for EM and UM populations each were used for microarray analyses, each with four technical replicas including dye swaps. Data were extracted using Agilent feature extraction software and raw data are available in NCBI's Gene Expression Omnibus (GEO) [44] under the accession number GSE45696 (subseries number GSE44193). Log_2_ratios were calculated using LOWESS normalized intensity values of UM and EM (log2 UM/EM) with R [45]. Subsequently, a manual filtering process was applied before statistical analysis, keeping only those oligonucleotides that were defined as representative (unique) for each gene (criteria “to be used in analysis”) according to the information provided together with the re-annotation of the array [43]. Remaining transcripts were submitted to a manual filtering process keeping only those transcripts that were present in all biological replicas, in at least three technical replicas of one biological replica in at least one condition (EM or UM). With these pre-selected data a statistical analysis for microarrays (SAM) [46] was performed using a one-class analysis. Significance cutoff was chosen at a FDR (false discovery rate) of 0.01, and only average log_2_ratios <−0.585 or >0.585 (which corresponds to a 1.5- fold difference in transcript levels) were defined as relevant. Genes fulfilling these requirements are described in the text as significantly differentially transcribed. The final analysis focused on oligonucleotides representing sense transcripts (although the array also contained oligonucleotides representing putative antisense RNAs for each corresponding gene locus).

### SuperSAGE

Sample collection for SuperSAGE equaled that for the microarray approach. Total RNA was extracted from whole worm batches of 50 males (EM or UM). RNA was quality-controlled on a denaturing formaldehyde gel as well as with a bioanalyzer (Agilent 2100 Bioanalyzer, Agilent Technologies). The following experimental procedure to perform SuperSAGE was done as described previously [47] with minor changes [48]. Raw data were deposited at GEO [44] under the accession number GSE45696 (subseries number GSE45628).

Tags were annotated applying the same procedure as for the re-annotation of the microarray [43] and separated or added up according to their annotation to the predicted exon or intron parts of a CDS in either sense or antisense orientation. Counts were normalized to a library size of 1,000,000, and a filtering process was applied keeping only those transcripts that were detected in two out of three biological replicas in EM or UM. A program implementing the statistical method of Audic and Claverie [49] using a Bio_Sage script (pearl) (http://search.cpan.org/~scottzed/Bio-SAGE-Comparison1.00/lib/Bio/SAGE/Comparison.pm) was used for significance analysis of the data. The statistical cutoff was p<1^−10^. As with the array, only transcripts with average log_2_ratios <−0.585 or >0.585 were selected for further analysis. For data comparison only annotated sense transcripts were used, while 7,124 transcripts annotated as antisense as well as 19,610 tags without annotation were excluded from analysis.

### SuperSAGE and microarray data comparison

Data for sense transcripts from SuperSAGE and microarray were comparatively analyzed using Spotfire [50] and Microsoft-Excel. Smp_numbers without a match in both analyses were manually checked again. The same approach was used for comparative-analysis to other data. An intersection data-set was created, which contained only transcripts detected by both analyses.

### Real-time PCR

These were performed on a Rotor Gene Q (Qiagen) using SYBR-Green MasterMixes (PerfeCTa SYBR Green SuperMix (Quanta) or RotorGene SYBR Green PCR Kit (Qiagen)). PCRs were performed in a total volume of 20 µl, with a three-step thermo-profile and a final melting-curve analysis. Primers, their concentrations, annealing temperatures and efficiencies are listed in Supplementary Table S1. All primers were synthesized by Biolegio (Netherlands). Primer-efficiencies were determined with a standard-curve on diluted gel-eluate with 1∶10 dilution steps [51]. Efficiencies were considered to be optimal between 85–100%. RNAs from EM and UM were evaluated for similar quality on denaturing formaldehyde-agarose (1.2%) gels. cDNAs were diluted 80-fold for usage and added 1∶4 to the final reaction. Absolute quantification was achieved by including the standard curve in each run [52]. Fold changes (EM/UM) were calculated using UM as calibrators [52]. To facilitate comparisons to microarray and SuperSAGE data log_2_-values of the fold changes were determined as previously described [53], [54]. Standard-curves were performed in duplicate after initial tests for primer concentrations, and reactions on cDNA-samples were performed in triplicates. The significance of individual experiments was checked applying the “Exact Wilcoxon rank sum test” using the exactRankTests package for R [45]; [53]–[57]. Correlation of real-time PCR and transcriptome data was checked with the Spearman's correlation coefficient [53].

### Stage- and organ-specific RT-PCRs

For stage-specific detection of SmFst transcripts, cDNAs from EM, UM, EF, UF, miracidia, and cercariae were generated and tested in standard PCR-reactions with primers for SmFst (fwd-5′- TGTTGTAAACGTGGTGGATTC-3′ and rev-5′-CGACATTfTGCATTTTGGTTC-3′) and primers for actin (fwd-5′-GGAAGTTCAAGCCCTTGTTG-3′ and rev-5′-TCATCACCGACGTAGCTGTC-3′) as positive control. PCR-products were separated on a 2% agarose gel.

To obtain organ-specific RNA a recently established protocol was used [58]. In short, adult schistosomes (about 50 individuals) maintained in M199-medium at RT were transferred to reaction vessels containing 500 µl of tegument solubilisation (TS)-buffer (0.5 g Brij35 (Roth), 0.5 g Nonidet P40-Substrate (Fluka), 0.5 g Tween80 (Sigma), and 0.5 g TritonX-405 (Sigma) per 100 ml PBS (137 mM NaCl, 2.6 mM KCl, 10 mM Na_2_HPO_4_, 1.5 mM KH_2_PO_4_ in DEPC-H_2_O, pH 7.2–7.4)). Following incubation at 37°C in a thermal shaker (TS-100, Biosan) at 1,200 rpm for 5 min to solubilise the tegument, the musculature was digested by protease treatment. To this end 500 µl elastase-containing medium (Sigma, #E0258; freshly dissolved in non-supplemented M199-medium, 5 units/ml) were used and the worms slightly agitated (600 rpm) in the thermal shaker at 37°C for 30–40 min. Progress of digestion was monitored by microscopic inspections using 20 µl aliquots. Upon the start of tissue fragmentation, reproductive organs such as ovary and testes were liberated. 1 ml non-supplemented M199-medium was added and the content of the vessel decanted to Petri dishes for manual collection of testes and ovaries, which were identified by their characteristic morphologies. If necessary, purification of the gonad tissue from residual parenchyma tissue was achieved by repeatedly collecting and transferring the organs to further Petri dishes containing 2 ml of non-supplemented M199-medium. Finally, the organs were collected using a 10 µl-pipette, transferred to 1.5 ml-tubes, and concentrated by centrifugation for 5 min at 1,000 g, and 1 min at 8,000 g. Following removal of the supernatant, the gonads were immediately frozen in liquid nitrogen and stored at −80°C for further use.

Total RNA was extracted from the organs as described before [58] using the PeqGOLD TriFast reagent (Peqlab; 500 µl TriFast-solution per extraction of 50 testes or 50 ovaries), and the resulting RNA pellet was resuspended in 10 µl DEPC-H_2_O each. RNA quality and quantity were checked by electropherogram analysis (Bioanalyzer 2100; Agilent Technologies). RT-PCRs were basically performed as described above (standard PCRs) using the following primer combinations to amplify gene transcripts of SmFST (fwd-5′-GAACCAAAATGCAAATGTCG-3′; rev-5′-GCCATGATTGTTCATTCCA-3′), SmBMP (q51- fwd-5′- GTCAAAATGAACAAAATCA-3′; q51- rev-5′- GTTACGTCGAACACTTTG-3′), and SmInAct (q1b-fwd-5′- CACAATTTGGTAATGTTCAACG-3′; q1b-rev-5′- AACTACAAGCACATCCTAAAACAA-3′).

### *In situ*-hybridization

Localization experiments were performed as previously described [59] with the following modifications: hybridization temperature was 42°C, and slides were washed up to 1× SSC. Two different probes were used for detection of SmFst transcripts: probe 1 was 571 bp long (position 208–778), while probe 2 was 306 bp long (position 914–1219). The probe for SmInAct was 440 bp long (position 206–646). Two probes were designed for SmBMP detection: probe 1 had a length of 455 bp (position 2240–2694) and probe 2 was 565 bp long (position 1041–1605).

### Yeast two-hybrid experiments

For SmFst–SmInAct/SmBMP interaction studies, yeast two-hybrid (Y2H) assays were performed. To this end full-length SmFst was cloned into the Gal4-BD vector pBridge using the following primers, which were designed according to the sequence information available at SchistoDB 2.0 [60] for Smp_123300: fwd-5′-*GAATTC*ATGGAAGAGAGTATATCACAATTAG-3′ (italics: cleavage site for *Eco*RI), rev - 5′-*GTCGAC*TTAGAATAAATTTGAATATTTTCC-3′ (italics: cleavage site for *SalI*). Full-length SmInAct was cloned into the Gal4-AD vector pACT2, using the following primers: fwd-5′-*CCCGGG*GATGAATAGAATGTTTAAATTAATAAAAC-3′ (italics: cleavage site for *Sma*I), rev-5′-*CTCGAG*TTAACTACAAGCACATCCTAAA-3′ (italics: cleavage site for *Xho*I). Due to its large size, the sequence for SmBMP was split into four sub-fragments, which were separately cloned into pACT2. The following primers were used: SmBMP-Y2H-Cterm-5′- 5′-*CCCGGG*GAAACCAAGATCAATTAATTATCCTAAC-3′, SmBMP-Y2H-ncbi-5′– 5′-*CCCGGG*GATGAACTCAAATATTTTAACAAAATCAG-3′, SmBMP-Y2H-ncbi-Nterm-5′ – 5′-*CCCGGG*GATGGAAACAGAAAAGACAAAAC-3′, SmBMP-Y2H-overlap-5′ – 5′-*CCCGGG*TGAAATAAATAGTACATCATTCTACTGG-3′ (italics: cleavage site for *Sma*I); SmBMP-Y2H-Cterm-3′ – 5′-*CTCGAG*TTAACGACAAGCACAACTTTC-3′, SmBMP-Y2H-db-Nterm-3′ – 5′-*CTCGAG*AATTGCTTACATTATTATTATTCAGAGG-3′, SmBMP-Y2H-ncbi-Nterm-3′ – 5′-*CTCGAG*GTTCTTTAGATGGTTTTCGTATATTATC-3′, SmBMP-Y2H-overlap-3′ – 5′-*CTCGAG*GATGATTATTTGTTTGTAATACATTTG-3′ (italics: cleavage site for *Xho*I). PCR products were separated on 1.0% agarose gels. The amplicons were cut out from the gel, and the DNA extracted using the PeqGold Gel Extraction Kit (Peqlab) following the manufacturer's protocol. Extracted fragments were cloned into pDrive (Qiagen) and later regained by restriction-digestion to be again checked for correct size on a 1.0% agarose gel and extracted. Finally, fragments were ligated into pBridge and pACT2, respectively, using T4 Ligase (Promega). Sequences were checked for integrity and a correct ORF by commercial sequencing (LGC Genomics, Berlin).

The SmFst-containing plasmid was transformed in to yeast cells (AH109) together with either one of the other plasmids prepared for the interaction studies. To control successful transformation yeast clones were grown on selection plates (SD-Trp/-Leu/-His/-Ade). β-galactosidase (β-gal) liquid- and filter- assays were performed to confirm interactions (Yeast protocols handbook, Clontech).

### *In silico* analyses

The following public domain tools were used: SchistoDB (http://www.schistodb.net/schisto/; [60]), BLASTx (http://www.ncbi.nlm.nih.gov/BLAST), restriction mapper version 3 (http://www.restrictionmapper.org/). Data were analyzed for enriched genes of the ontology categories with Ontologizer [61] using contig annotations, which were the basis for the microarray design [41], [42]. Furthermore, only sense-orientated genes/transcripts were used for this analysis. Network enrichment analyses were done with the Ingenuity Pathway Analysis (IPA) tool (http://www.ingenuity.com; [62]). Only those transcripts with homology to a human molecule >60% and an e-value<10^−10^ were used, as defined during the re-annotation of the microarray [43]. For SuperSAGE-detected transcripts the according information was obtained from the same source as far as available. For allocation of transcripts to predicted *S. mansoni* metabolic pathways the function “omics viewer” of the software tool SchistoCyc was used (available at SchistoDB 2.0; [59]. The online-tool SMART (http://smart.embl-heidelberg.de/; [63], [64]) was used to predict protein domains.

### List of *S. mansoni* genes mentioned in the text

Smp_135230 - dopa decarboxylase; Smp_145140 - wnt5A; Smp_155340 – frizzled; Smp_036470 - oxalate-formate antiporter; Smp_135020 - oxalate-formate antiporter; Smp_169190 - tegument protein; Smp_161500 - rhodopsin-like orphan GPCR; Smp_131110 - p14; Smp_000270 - fs800-like; Smp_000430 - ‘eggshell precursor protein’; Smp_00280 - fs800-like transcript; Smp_123300 (KC165687) – *S. mansoni* follistatin; Smp_090140.2 - Ftz-F1 interacting protein; Smp_090520 - purin nucleoside phosphorylase; Smp_123010 – cationic amino acid transporter; Smp_095360.x – fatty acid binding protein; Smp_065580.x – heterogeneous nuclear ribonucleoprotein k; Smp_033950 - Smad4; Smp_144390 - *S. mansoni* activin receptor; Smp_049760 – TGFβRI; Smp_093540.3 – ActRI; Smp_124450 - ActRI/BMPRIa; Smp_080120.2 – ActRIIa; Smp_144390 - ActRIIb.

## Results

### Generation and comparative analyses of pairing-dependent transcription profiles of *S. mansoni* males

To produce a comprehensive data set of genes differentially transcribed between EM and UM, two methods were chosen. Because both methods have successfully demonstrated their capacities in the past, microarray and SuperSAGE analyses were applied in parallel to generate complementary data sets of genes differentially transcribed between EM and UM. For microarray analyses a *S. mansoni*-specific 60-mer oligonucleotide microarray platform was used, which represents nearly the complete genome of *S. mansoni* [42], [43]. The platform-independent SuperSAGE represents a technically improved modification of ‘serial analysis of gene expression’ (SAGE) by generating 26 bp sequence tags of all sample mRNAs containing a *Nla*III restriction site by a high-throughput sequencing approach [47]. Combining both methods, we expected to produce data sets complementing each other and providing independent indications for the importance of particular transcripts. To confirm differential transcription of genes from an expected overlap, or from individual data sets outside this overlap, we additionally performed real-time PCR experiments for selected candidates.

In order to facilitate the interpretation of the large scale transcriptome data and selection of molecules for first characterization studies, gene ontology (GO) analysis as well as two further analyses tools, Ingenuity Pathway Analysis (IPA) [62] and the metabolomics tool SchistoCyc [60] were applied. IPA operates with a curated biological knowledge base from the literature to generate molecular networks enriched for proteins encoded by significantly regulated genes from large-scale data sets. Similarly it searches for canonical pathways and predicts the activation of transcription factors. The SchistoCyc function ‘omics viewer’ allocates an uploaded set of genes to predicted *S. mansoni* metabolic pathways.

### Microarray-analyses revealed 526 genes to be differentially transcribed

**Figure 1:**
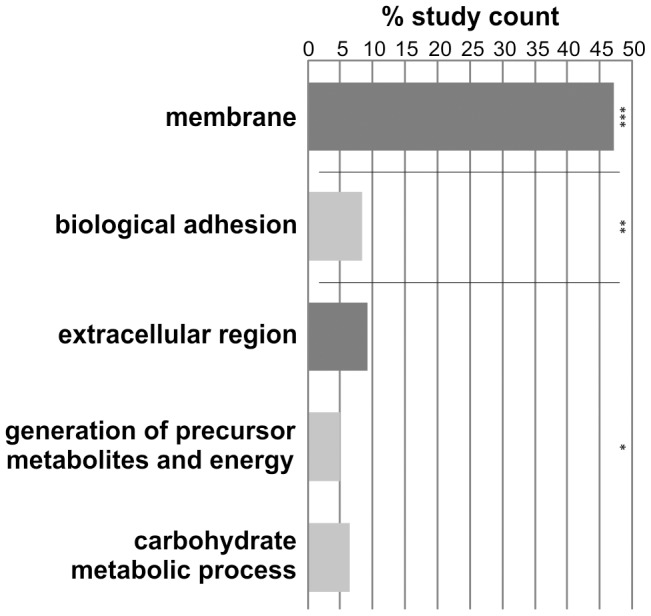
GO for genes differentially regulated according to the microarray analysis. This analysis was performed for genes significantly and differentially transcribed between EM and UM. For transcripts up-regulated in EM, no significantly enriched GO-categories were found. Shown are those categories significantly enriched (% study count) within the data set of genes up-regulated in UM. Light grey: biological process; Dark grey: cellular component; p-values for the GO-analysis: *p<0.05, ** p<0.005, *** p<0.0001.

The results of the microarray analysis were extracted and evaluated with Agilent feature extraction software. Subsequent data-processing included calculation of log_2_ratios [53], [65], data-filtering for consistency of transcript detection, and annotation of detected transcripts. After re-annotation of the 44 k oligo array in 2011 [43], 19,197 oligonucleotides were selected as gene representatives: 11,132 detecting RNA in orientation of the predicted transcript, 8,065 detecting RNA complementary to the predicted transcript. Following hybridization and data processing, 10,115 of these representatives were detected as transcribed, 1,966 representing putative antisense and 7,494 representing sense messages. Significance analysis of microarrays (SAM) [46] was performed for all transcripts, and only sense transcripts were analyzed further. Of these, 526 transcripts were significantly differentially transcribed; 229 were up-regulated in EM and 297 up-regulated in UM. Gene ontology (GO) analyses were performed in order to get an overview about the categories these genes could be assigned to. Interestingly, enriched categories were only found for genes up-regulated in UM (Fig. 1).

### SuperSAGE revealed 253 transcripts to be differentially transcribed

Again, three samples of EM and UM were collected each, independently from each other as well as from the microarray samples. RNA was extracted from 50 worms per sample and quality-checked. The further procedure was executed by GenXPro according to an internal protocol [47], [48]. SuperSAGE-tags were annotated in the same way as the revised version of the 44 k oligo array [43], and counts for different tags were summed up if they had shown the same annotation. Similar to the microarray data-analysis, transcripts were classified into sense- and antisense orientation relative to the protein-coding gene in a given locus. Additionally, SuperSAGE-transcripts were further distinguished between predicted intron- or exon-sequences. Thus four categories of SuperSAGE-detected tags were defined, and accordingly up to four transcripts could represent one gene. This explains the large number of 25,597 gene-specific transcripts detected with SuperSAGE, which exceeds the assumed number of *S. mansoni* genes more than twice [33]. Statistics were performed according to the method of Audic & Claverie [49]. Subsequent to this significance analysis, 5,987 transcripts without annotation and 7,124 transcripts classified as antisense were excluded from further analyses. Of the remaining 12,486 sense transcripts, 8,969 were classified as exon and 3,517 as intron sequences. Taking out redundancy for genes represented by an exon as well as an intron sequence, sense transcripts were found for a number of 9,344 unique genes. Of these, 5,601 genes had representatives in both groups exons as well as introns. For 2,581 genes only exon-representing sense transcripts were detected, and for 219 genes only intron-representing sense transcripts were detected.

Selected from these were candidates showing a normalized detection value of at least 10 tags in at least one library and significantly differential regulation between EM and UM. These criteria were met by 253 transcripts. Of these 218 were up-regulated in EM and 35 in UM. GO analyses showed enriched categories neither for the transcripts up-regulated in EM nor for those up-regulated in UM.

### Data-comparison

**Figure 2:**
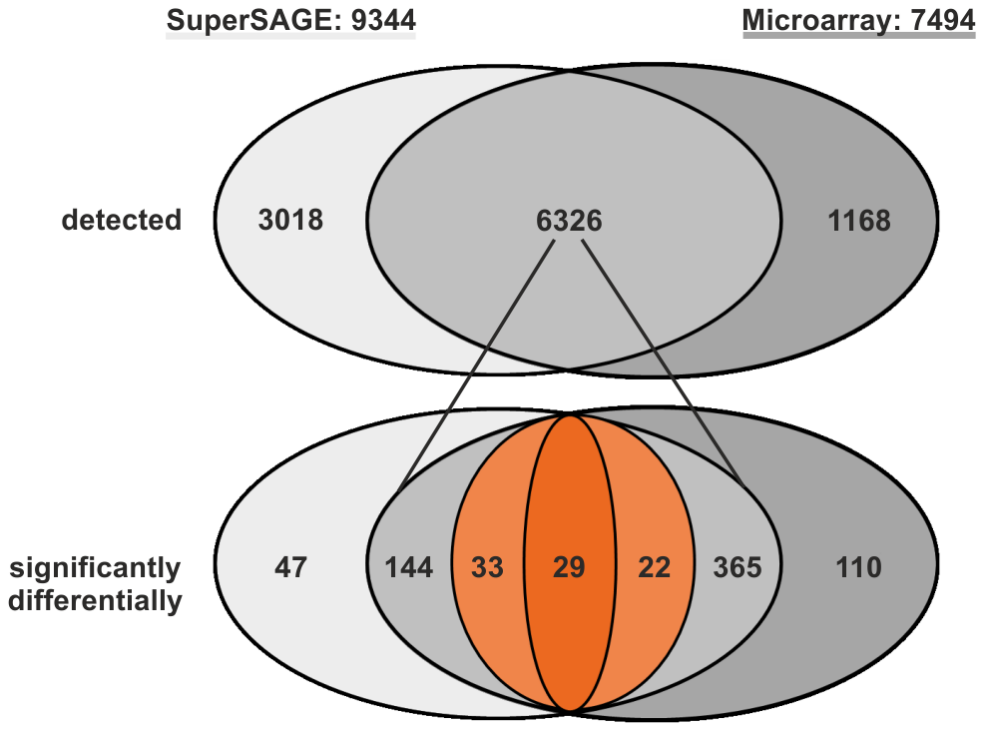
Venn diagram of SuperSAGE and microarray data analyses. For final analyses we selected 9344 and 7494 genes for which sense transcripts were detected by SuperSAGE and microarray, respectively. The upper diagram (detected) presents numbers of genes, whose transcripts were identified by either one (light grey, left: SuperSAGE, 3018; dark grey, right: Microarray, 1168) or both methods (intersection; grey, middle part, 6326). The lower diagram (significantly differentially) presents genes, whose transcripts were significantly differentially (Super SAGE, p<1^−10^; Microarray, q<0.01; log_2_ratios <−0.585 or >0.585) regulated between EM and UM according to at least one analysis (outside the intersection: light grey, left: SuperSAGE, 47; dark grey, right: microarray, 110; within the intersection: grey, left: 144 found as differentially transcribed according to SuperSAGE only; grey, right: 365 found as differentially transcribed according to microarray data analysis only; light orange, left, 33: found as significantly regulated by both analyses but differentially transcribed only according to SuperSAGE; light orange, right, 22: found as significantly regulated by both analyses but differentially transcribed according to microarray data analysis only). Finally, a number of 29 transcripts (dark orange, middle) were found representing genes being differentially transcribed according to the analyses of the data sets obtained by both methods.

Comparing sense transcripts detected by microarray and SuperSAGE, 6,326 transcripts were found by both methods, while additional 3,018 and 1,168 were exclusively detected by SuperSAGE or microarray, respectively (Fig. 2). The number of counts representing genes being differentially transcribed between EM and UM varied between the methods used. This was influenced by the underlying, method-specific statistics leading to subsets of counts that were significant for only one of the two data sets although the same transcripts were also present in the other data set (Fig. 2). The stringent analysis criteria for differential regulation were met according to both approaches by 29 transcripts. Among these were genes with various biological functions such as metabolism (NAD-dependent epimerase/dehydratase, sodium dicarboxylate cotransporter, fatty acid acyl transferase-related, oxalate-formate antiporter), neurotransmitter synthesis (aromatic amino acid decarboxylase), enzyme activity (kunitz-type protease inhibitor), microfilament organization (villin, nebulin), membrane dynamics/vesicle formation (endophilin b1, snf7-related), molecular interaction/communication (surface protein PspC, cadherin), calcium metabolism (sarcoplasmic calcium-binding protein), chromatin organization (histone h1/h5), signal transduction (pinch, dock, follistatin), and others less well defined (cancer-associated protein gene, loss heterozygosity 11 chromosomal region 2 gene) (Table 1).

**Table 1:** Selected genes significantly differentially transcribed according to both analyses.

Gene	Gene annotation	M-log_2_(EM/UM)	S-log_2_(EM/UM)	S-tag
Smp_135230	aromatic-L-amino-acid decarboxylase	2.58	2.08	Exon
Smp_168940	NAD-dependent epimerase/dehydratase	2.66	1.49	Exon
Smp_052230	kunitz-type protease inhibitor	1.83	1.78	Exon
Smp_008660.x	villin	0.94	1.43	Exon
Smp_163720	endophilin B1	0.83	1.40	Exon
Smp_055220.x	surface protein PspC	0.83	1.19	Exon
Smp_020540.x	pinch	0.61	1.10	Exon
Smp_040560	cancer-associated protein gene	0.85	1.02	Exon
Smp_073130.x	loss of heterozygosity 11 chromosomal region 2 gene, a protein homolog (mast cell surface antigen 1) of masa-1	0.74	1.00	Exon
Smp_170080	sodium/dicarboxylate cotransporter-related	1.18	0.92	Exon
Smp_080210	lipid-binding protein	0.65	0.89	Exon
Smp_151490	nebulin	0.80	0.89	Exon
Smp_070030	snf7-related	0.71	0.79	Exon
Smp_003770	histone h1/h5	0.86	0.68	Exon
Smp_010770.x	fatty acid acyl transferase-related	−1.71	−0.89	Exon
Smp_123080	sarcoplasmic calcium-binding protein (SCP)	−0.86	−1.00	Exon
Smp_123300	**follistatin (SmFst)**	−2.15	−1.23	Exon
Smp_036470	oxalate-formate antiporter	−2.91	−1.76	Exon
Smp_158480	AMP-dependent ligase	2.82	3.55	Intron
Smp_151620	cadherin-related	0.63	1.41	Intron
Smp_135520	dock	−1.31	−0.65	Intron

Of all transcripts detected in the SuperSAGE and microarray analyses, 29 were differentially transcribed according to both methods. Of these, 21 with functional annotations are presented here, excluding those, which were annotated as ’hypothetical protein’. Follistatin (Smp_123300) is highlighted (see also text). Genes, gene annotations, log_2_(EM/UM) ratios of microarray analyses (M- log_2_) and SuperSAGE (S-log_2_) as well as exon/intron presence of the SuperSAGE tags (S-tag) are given.

Differentially transcribed genes from either microarray or SuperSAGE were assigned to predicted *S. mansoni* metabolic pathways using the “omics viewer” function of SchistoCyc [60] (Supplementary Table S2). Comparing the results of these analyses, transcripts coding for enzymes involved in carbohydrate metabolic processes, citrate cycle, aerobic respiration and amino acids metabolic processes were mostly down-regulated in EM compared to UM. For base metabolic processes, transcripts for enzymes involved in synthesis were rather down-regulated in EM, while transcripts for enzymes involved in degradation and salvage pathways were up-regulated in EM. Several transcripts with different directions of regulation were found for enzymes participating in lipid and fatty acid metabolic processes. Interestingly, data sets for differentially transcribed genes contained none coding for enzymes related to pentose-phosphate cycle or glycolysis. Only one enzyme within the SuperSAGE/microarray-intersection of differentially transcribed genes was allocated to metabolic pathways, aromatic amino acid decarboxylase (dopa decarboxylase, DDC, Smp_135230/NP_001076440.1). DDC was up-regulated in EM and allocated to phenylethanol and catecholamine biosynthesis. This indicates a pairing-dependent adaptation for usage of neurotransmitters like dopamine can be assumed.

IPA was performed for the data-overlap of transcripts differentially regulated according to both transcriptome analyses. While no significantly enriched canonical pathways were detected, one significantly enriched network was found, namely ‘embryonic development, hair and skin development and function, organ development’. It contained nine differentially transcribed genes, seven up-regulated in EM and two up-regulated in UM, including SmFst (Supplementary Table S2, Supplementary Figure S1). Thus IPA additionally highlighted SmFst and a putative differential regulation of neurotransmitter synthesis through DDC.

Looking for explanations why transcripts were detected by one method only without complementary counterpart, these specific transcript groups were selectively analyzed in more detail. Out of the 47 transcripts differentially regulated in the SuperSAGE-only group, oligonucleotides existed on the microarray for 25 out of 47, thus 22 transcripts were newly detected by SuperSAGE (Table 2). Next, transcripts within the microarray-only group (110) were analyzed for the presence of *Nla*III restriction-enzyme recognition sites. cDNA synthesis and *Nla*III restriction are the first two steps during the SuperSAGE procedure. Transcripts lacking the restriction site or with an *Nla*III restriction too close to the polyA-tail will not be detected. As a snap sample of 110 candidates of the microarray-only group, we selected 10 which not only had a Smp_number but also a functional annotation other than ‘hypothetical protein’. Of these 8 had *Nla*III restriction sites within their predicted CDS (Table 3). Thus indeed, the existence of *Nla*III restriction may have influenced the detection of transcripts by SuperSAGE. Also other methodological differences may have contributed to the resulting number of transcripts detected by both methods, although not as differentially transcribed in both analyses. While in microarray experiments light intensities are measured, during SuperSAGE the number of transcripts is counted, resulting in different log_2_ratios (some below the selected threshold of log_2_ratio = 0.585). Also the number of technical replicas differed. While three biological replicas without technical replicas were used for SuperSAGE, four technical replicas for each biological one were done in case of the microarrays, which is part of the methodological procedure. Furthermore, different statistics had to be used for the analyses of the results from both methods, which influenced the outcome with regard to the significance of the differences detected between EM and UM.

**Table 2:** Selection of transcripts detected by SuperSAGE only.

Gene	Gene annotation	S-tag	Log_2_(EM/UM)	p-value	Probe
Smp_130690	zinc-finger protein, putative	Exon	8.54	1.59 e^−20^	Q2_P01072
JAP09507.C	dynein, axonemal, heavy chain 10 [*Homo sapiens*]	Exon	5.57	1.98 e^−246^	Q2_P39947
Smp_011100	M16 familypeptidase, putative	Exon	4.30	5.49 e^−38^	n.o.
Smp_157750	RNA-binding protein, musashi-related	Exon	3.31	8.37 e^−11^	Q2_P20135
Smp_173350.x	egg protein, CP391S-like	Exon	2.70	6.52 e^−23^	Q2_P18640
Smp_008660.1	gelsolin isoform precursor [*Homo sapiens*]	Exon	0.91	0	Q2_P18327
Smp_160360	putative sodium/chloride-dependent neurotransmitter transporter	Exon	0.89	2.08 e^−26^	Q2_P00318
Smp_097020.4	fbxl20, putative	Exon	0.74	2.76 e^−11^	Q2_P24759
Smp_186050	heat-shock protein, putative	Exon	0.73	2.02 e^−141^	Q2_P34228
Smp_163320.x	ank repeat-containing, putative	Exon	0.68	3.28 e^−28^	Q2_P10446
Smp_013860	glutamate-cysteine ligase	Exon	0.63	2.63 e^−29^	Q2_P05616
Smp_140450.1	cleavage and polyadenylation specificity factor, putative	Exon	0.60	5.33 e^−12^	Q2_P30180
Smp_012380	potassium channel beta, putative	Exon	−0.76	4.42 e^−12^	Q2_P10250
Smp_164480	tubulin beta chain, putative	Exon	−0.83	1.05 e^−43^	n.o.
Smp_124570	leucine zipper protein, putative	Exon	−0.92	2.92 e^−16^	Q2_P05434
Smp_170020	neuropeptide receptor, putative	Intron	−0.93	3.21 e^−18^	n.o.
Smp_152630	MEG-12	Exon	−0.96	1.79 e^−178^	Q2_P14896
Smp_068240	zinc-fingerprotein, putative	Exon	−1.05	7.70 e^−30^	n.o.
Smp_105730	CAI-2 protein, putative	Exon	−1.07	1.88 e^−39^	n.o.
Smp_000170	neurocalcin homolog (drosnca), putative	Exon	−1.15	4.11 e^−29^	Q2_P32680
Smp_159950	neuropeptide Y precursor, putative	Exon	−1.80	2.68 e^−11^	n.o.
Smp_098780	forkhead protein domain	Exon	−4.85	0	n.o.
JAP10881.C	mucolipin 2 [*Homo sapiens*]	Exon	−5.21	6.26 e^−294^	Q2_P41336

Of the 47 genes differentially transcribed between EM and UM within the SuperSAGE-only group, 23 had Smp_numbers and functional annotations other than ‘hypothetical protein’. Respective oligonucleotide representatives on the microarray are noted. n.o.: no oligo representing this gene is included on the microarray. Genes, gene annotations, exon/intron presence of the SuperSAGE tags (S-tag), log_2_(EM/UM) ratios, p-values, and microarray probe numbers are given.

**Table 3:** Selection of transcripts detected by microarray only.

Gene	Gene annotation	Log_2_(EM/UM)	q-value	*Nla*III
Smp_169190	tegumental protein, putative	1.80	0.000	yes
Smp_033000	calcium-binding protein, putative	1.28	0.000	no
Smp_135020	oxalate-formate antiporter, putative	1.18	0.000	yes
Smp_133660	lin-9, putative	1.07	0.002	yes
Smp_173240	cement precursor protein 3B variant 3, putative	0.85	0.001	yes
Smp_081140	dbl-related	0.79	0.002	yes
Smp_195010	HMG-CoA synthase	−0.64	0.000	yes
Smp_053120	S-adenosyl-methyltransferase mraW, putative	−0.69	0.000	no
Smp_024290	MAP kinase kinase protein DdMEK1, putative	−0.70	0.002	yes
Smp_103360	protein kinase	0.78	0.002	yes

Shown are 10 of 110 genes, which were detected only in the microarrays and found to be significantly and differentially transcribed. Smp_numbers and annotations other than ‘hypothetical protein’ existed for these 10 genes only. Genes, gene annotations, log_2_(EM/UM) ratios, q-values and presence or absence (yes/no) of *Nla*III restriction enzyme recognition sites are indicated.

### Real-time PCR experiments confirmed differential transcription of selected genes

For a sub-set of 21 transcripts we performed real-time PCR experiments (i) to confirm the results obtained in the combinatory analysis, and (ii) to confirm those microarray or SuperSAGE data, for which no complementary results existed. With respect to their putative biological relevance, transcripts for signal transduction molecules, surface molecules, metabolism-associated proteins and transcription factors were chosen for verification (Supplementary Table S1). Additionally, the regulation of three transcripts for egg-shell precursor proteins was analyzed. A Wilcoxon rank sum test for real-time PCR data detected significant differences between EM and UM transcript levels for most of the differentially transcribed genes present within the overlap of microarray and SuperSAGE data (Supplementary Table S1). The test also confirmed significant differences between the two male stages for two genes detected by only one of the transcriptome studies. Using the Spearman's rank correlation coefficient, overall results from real-time PCR were compared to those from either one of the two transcriptome approaches. Data correlated significantly (p<0.01) between real-time PCR and microarray (r = 0.676) or SuperSAGE (r = 0.621) each.

**Figure 3:**
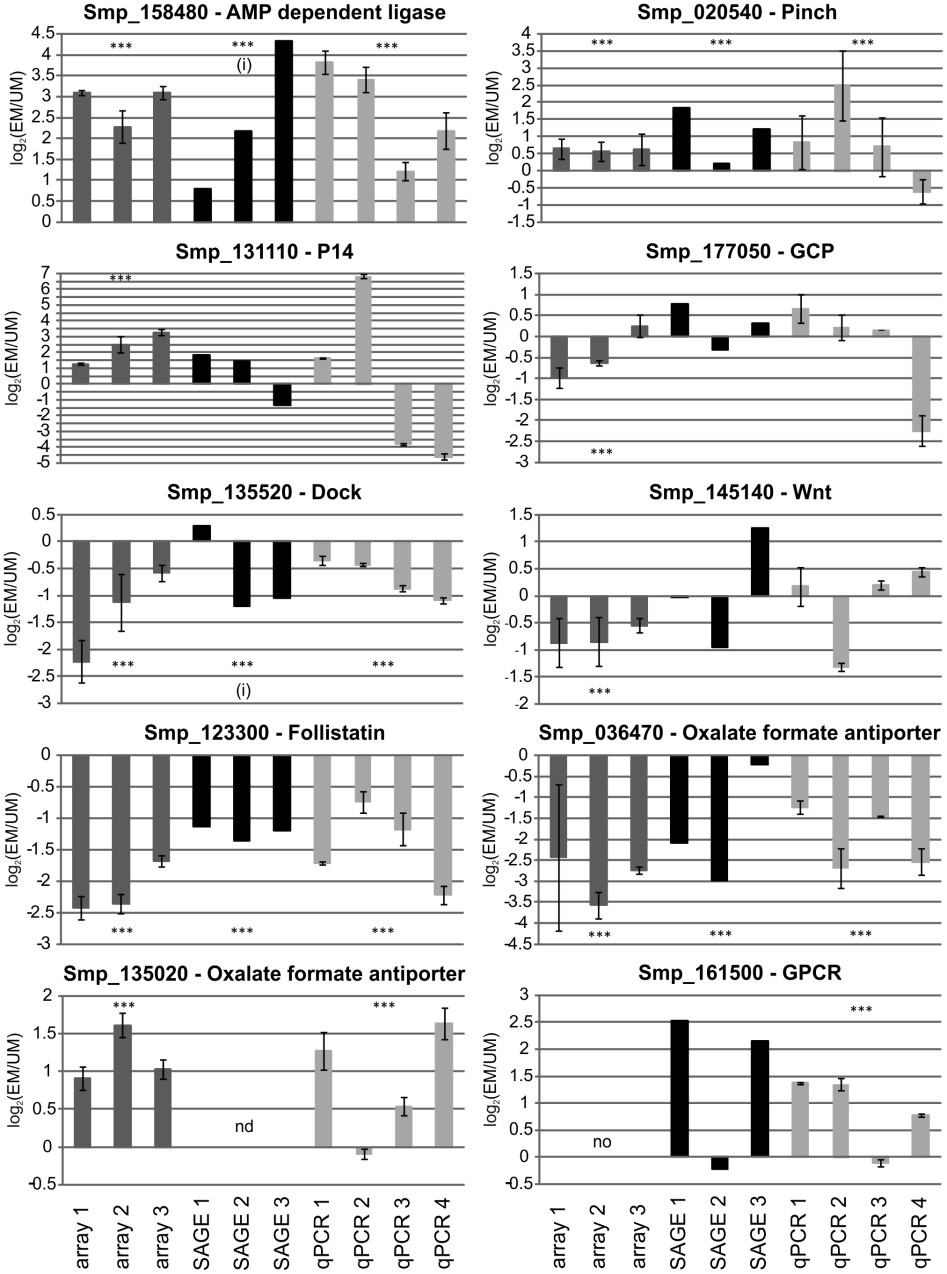
Exemplary results from real-time PCR analyses. Represented results are sorted according to technical categories. Log_2_ratios (EM/UM) are given for all biological replicas tested in either microarray, SuperSAGE or real-time PCR. grey: microarray; black: SuperSAGE; light gray: real-time PCR. Stars in graphs indicate significance in the according analysis. nd: no transcripts for this gene detected in this analysis; no: this gene is not represented by an oligo on the microarray; i: detected transcripts were aligned to a predicted intron. To ease comparison between graphs all y-axis intervals were set to 0.5.

Representatives for signal transduction-associated transcripts were SmFst, dock, and pinch (Figure 3; Supplementary Table S1). For all three genes transcription regulation was confirmed by real-time PCR. Comparing the results of all approaches, dock and pinch showed a slight biological variation within only one of the three analyses. In contrast, the transcriptional activity of SmFst was found to be consistently down-regulated without exception in EM (Figure 3), which substantiated the results of the transcriptome approaches. Since follistatins (FSTs) have regulatory function for TGFβ-signaling [39], [40], we additionally tested the transcriptional activities of further genes coding for members of TGFβ-pathways (Supplementary Table S1). In those cases where microarray or SuperSAGE indicated significant regulation for a TGFβ-pathways member, this finding was not confirmed by the complementary method or real time PCR.

Furthermore, we included wnt5A (Smp_145140) and one of its potential receptors, the seven transmembrane receptor frizzled (Smp_155340) into this analysis, since wnt-pathways are linked to developmental processes [66]. According to the microarray data Wnt5A and frizzled transcripts were significantly down-regulated in EM. The direction of regulation of both transcripts detected by SuperSAGE varied strongly between the biological replicates, and differences between EM and UM were not significant. This was confirmed by real-time PCR experiments (Figure 3, Supplementary Table S1), indicating that the wnt-pathway and/or associated members may not be essential with respect to EM/UM differences and are presumably influenced by additional biological parameters beyond pairing.

The AMP-dependent ligase attracted attention due to its uniform differential transcriptional regulation in both approaches and its putative function in DNA synthesis processes. Besides SmFst, it was one of the transcripts for which real-time PCR supported the previous findings, but in contrast to SmFst, transcription of the AMP-dependent ligase was strongly up-regulated in EM (Figure 3). A similar consistent result was obtained for one of the oxalate-formate antiporters (OxlT) (Smp_036470), whose transcript amount was lower in EM. In contrast, a second OxlT (Smp_135020) was detected as significantly up-regulated in EM by the microarrays only, which was confirmed by real-time PCRs. Further transcripts for membrane proteins analyzed in real-time PCR experiments were a tegument protein (Smp_169190), significantly up-regulated in EM according to the microarray analysis (Supplementary Table S1) and a rhodopsin-like orphan GPCR (Smp_161500) up-regulate in EM according to the SuperSAGE analysis (Figure 3). Both transcripts were not detected by the respective other transcriptome analysis. Real-time PCRs confirmed the presence and direction of regulation for both transcripts substantiating that microarray and SuperSAGE experiments can complement each other. Enhanced transcript levels of the rhodopsin-like orphan GPCR in EM were also confirmed by semi-quantitative real-time PCR [data not shown]. With respect to the fact that the physical contact between the genders stimulates differentiation processes in the female, tegumental proteins as such are potentially important for male-female interaction. Since GPCRs represent the biggest receptor class in schistosomes [66], and since they are known to be involved among others in regulating differentiation processes in diverse organisms [67]–[70], there may be candidates with roles in male-female interaction as well.

Surprisingly, transcript amounts for the egg-shell precursor protein p14 (Smp_131110) were significantly elevated in EM according to the microarray analysis. Our real-time PCR experiments, however, demonstrated strong variations between biological replicas, previously also indicated by SuperSAGE results. Analogous results were obtained for two other egg-shell precursor transcripts, fs800-like (Smp_000270), and ‘eggshell precursor protein’ (Smp_000430) (data not shown). Thus it seems obvious that egg-shell precursor protein transcripts can be strongly up-regulated in EM compared to UM, but this depends on the worm batch. Indirect support for this interpretation comes from a previous study, which provided evidence for another fs800-like transcript (Smp_00280) to be up-regulated in testicular lobes of paired males [71]. Other transcriptome studies, comparing EM and UM, also found transcripts of supposedly female-specifically expressed genes in males [37]. These findings can be explained by leaky expression control of such gender-associated genes, a phenomenon that can even lead to the development of female reproductive tissue in males as previously observed [11], [72]–[74].

### Sequence analyses of SmFst

The follistatin homolog SmFst appeared as one of the most interesting candidates for first functional characterization because of its consistent down-regulation in EM in all analyses, and its putative participation in schistosome TGFβ signaling processes. Based on the gene prediction (Smp_123300) obtained from the *Schistosoma* genome project, primers were designed to amplify its full-length cDNA. Following cloning and sequencing, minor differences were detected between SmFst (KC165687) and Smp_123300, revealing parts of predicted intron sequences as exon stretches (Supplementary Figure S2).

While other typical Fsts contain three follistatin-domains (FstDs) [75], SmFst encodes an open reading frame containing two, according to a SMART-domain analysis. FstDs are further subdivided into an EGF (epidermal growth factor)- and a Kazal (a protease inhibitor)-domain, which according to SMART do not follow each other directly in SmFst (amino acid positions: EGF-domains: 52–74, 312–337; Kazal-domains: 264–307, 398–434), as is the case for other Fsts [75]. The first EGF-domain of SmFst is located upstream of the first EGF-domain of other Fsts, and the second EGF-domain of SmFst is homologous to the third one of other Fsts. The first and second Kazal-domains of SmFst are homologous to the second and third Kazal-domains of other Fsts.

### SmFst is transcribed in miracidia and in adults with a preference for gonads

**Figure 4:**
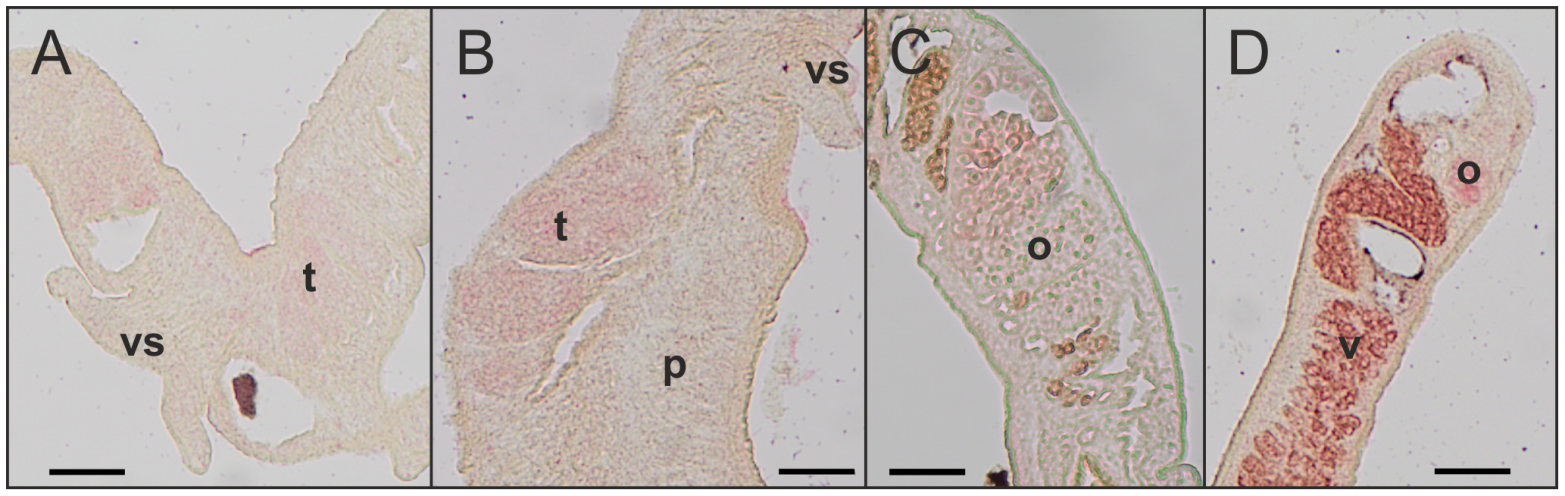
SmFst *in situ*-hybridization. *In situ*-hybridization experiments with sense and antisense probes resulted in variable SmFst signals, but always in the reproductive organs of couples and UM. A: UM, B: EM, C: EF, D: EF. t: testicular lobes, o: ovary, p: parenchyma, v: vitellarium, vs: ventral sucker. Scale bar: 50 µM.

To confirm the presence of SmFst in different life cycle stages of *S. mansoni* RT-PCR reactions were performed with RNAs from EF (pairing-experienced females), UF (pairing-unexperienced females), EM, UM and the free-living larval stages. SmFst transcripts were detected in all adult stages and miracidia, but not in cercariae. As positive control actin was amplified from all tested life cycle stages (Supplementary Figure S3). Localization studies by *in situ*-hybridization with couples as well as UM detected SmFst transcripts within the testicular lobes of both male groups and also in the vitellarium and ovary of female worms (Figure 4). Results concerning the detection of sense or antisense transcripts varied with probe sequence and probe batch. Organ-specific RT-PCR [58] confirmed the presence of transcripts for SmFst within ovary and testes (Supplementary Figure S4).

### SmInAct and SmBMP both localize in the reproductive organs and interact with SmFst

Fst was shown before to bind activin, which itself is an agonist of TGFβ-pathways [76], [77]. Although with lower affinity it also binds to a bone morphogenic protein (BMP), another agonist of TGFβ-pathways [78]–[81]. SmInAct, a S*chistosoma* activin-inhibin, was previously characterized [82] as well as SmBMP [83]. In our study, SmInAct transcripts were detected only by SuperSAGE but without differential regulation between EM and UM, which was also confirmed by real-time PCR results. SmBMP was down-regulated according to the microarray data, however, this was neither confirmed by SuperSAGE nor by real-time PCR. To obtain evidence on possible interactions between SmFst and SmInAct and/or SmBMP, co-localization and Y2H interaction studies were performed.

**Figure 5:**
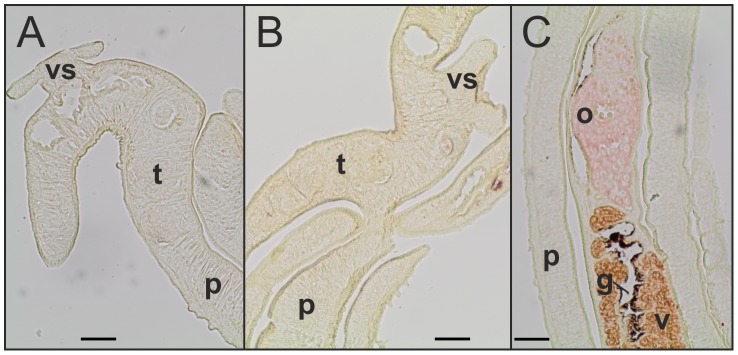
SmInAct *in situ*-hybridization. *In situ*-hybridization experiments detected sense and antisense transcripts in the ovary (o) of EF (C). No signals were obtained with either sense or antisense detecting probes for testicular lobes (t) of EM (A) or UM (B), or the vitellarium (v) (C). g: gut, p: parenchyma, vs: ventral sucker. Scale bar: 50 µM. Depicted results are representative for sense and antisense detecting probes.

**Figure 6:**
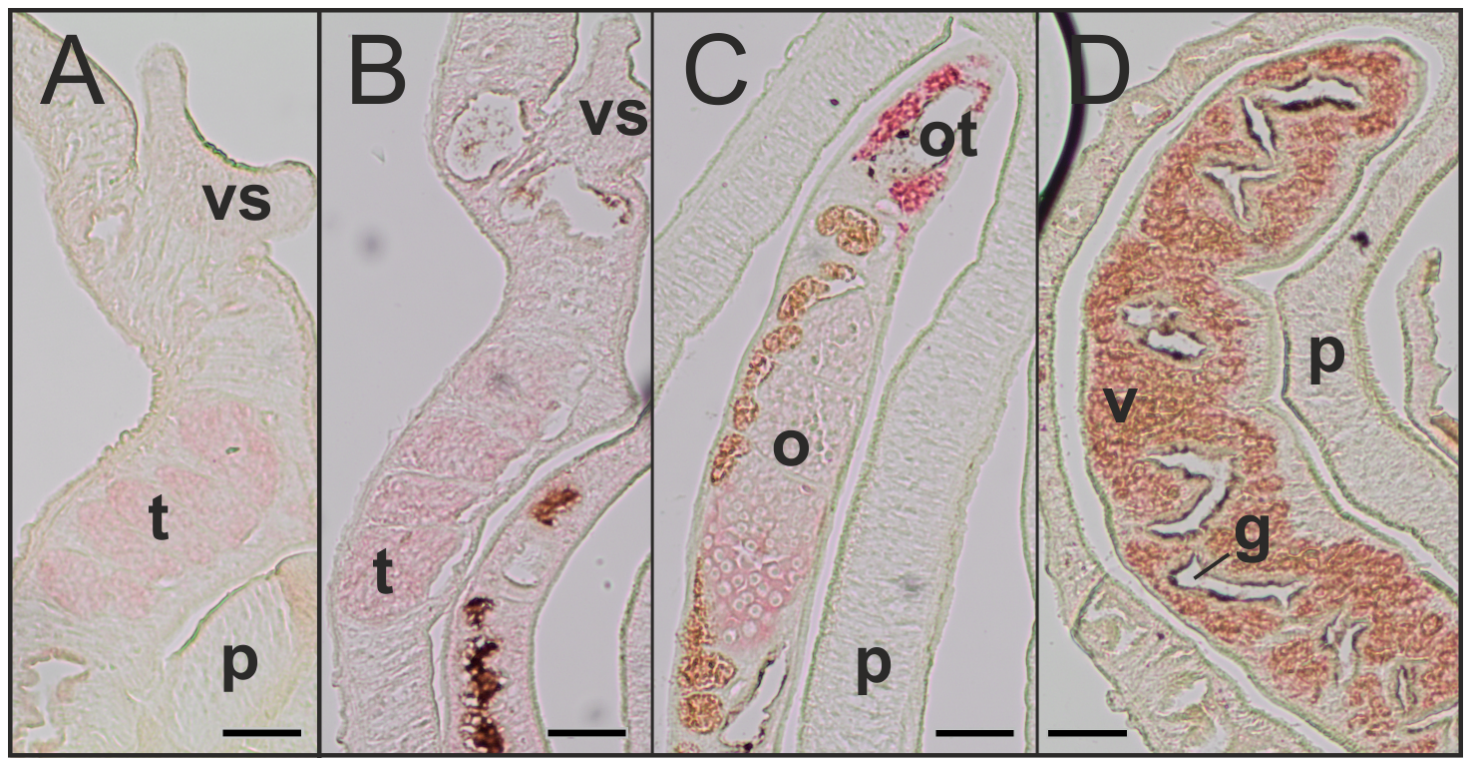
SmBMP *in situ*-hybridization. Both probes detected transcripts in the reproductive organs of couples and UM. Sense and antisense transcripts were detected. The pictures are representative examples for the detection of SmBMP transcripts, independent of probes and strand. A: EM, B: UM, C: EF, D: EF. t: testicular lobes, g: gut, o: ovary, ot: ootype, p: parenchyma, v: vitellarium, vs: ventral sucker. Scale bar: 50 µM.

Previous studies had localized SmInAct transcripts in the ovary and vitellarium of EF, without providing information on their presence in testicular lobes [82]. The results obtained in our study using two replicas with a hybridization probe based on the same sequence as used previously [82] showed sense and also antisense transcripts exclusively in the ovary of EF. No signal was detected within the vitellarium or testes of EM or UM (Figure 5). However, organ-specific RT-PCR on ovary and EM testes detected transcripts not only in the ovary but also in testes of EM (Supplementary Figure S4). For the localization of SmBMP two different probes were used. Transcripts were detected in all reproductive organs of couples as well as UM, but especially for probe 2 in the area around the ootype (Figure 6). Again, sense and antisense transcripts were detected, and results for EM-testes and ovary were confirmed by organ-specific RT-PCR (Supplementary Figure S4).

Even though the major interaction partner of Fsts is activin, it can also bind to BMP [79]. For males our *in situ*-hybridization experiments rather indicated physical proximity of SmFst transcripts to those for SmBMP, as both transcripts were detected within testicular lobes. In females, transcripts for all three molecules were detected within the ovary by *in situ*-hybridization, though organ-specific RT-PCRs also indicated the presence of SmInAct in male testes.

**Figure 7:**
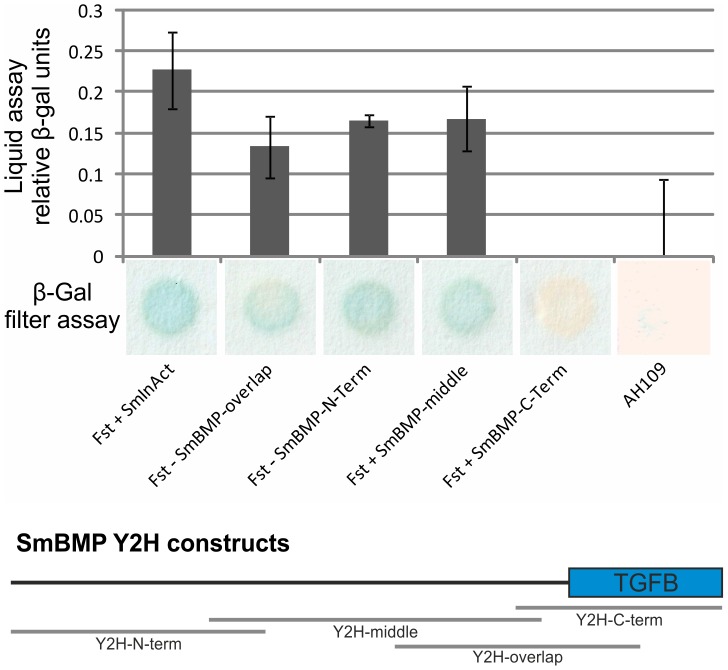
Yeast two-hybrid experiments with SmFst. Y2H β-galactosidase liquid- and filter-assays were performed with the full length sequences of SmFst and SmInAct, as well as full length SmFst and four different sequence parts of SmBMP (bottom part of diagram). The upper part shows results from β-gal liquid-assay, with interaction-values normalized to that of AH109, the lower part those from a representative β-gal filter assay. SmFst interacts with all tested interaction partners except the one containing the C-terminal part of SmBMP.

To provide evidence for protein interaction between SmFst and SmBMP or SmInAct, Y2H interaction studies were performed. To this end full-length SmFst was cloned into the Gal4-BD vector pBridge, while full-length SmInAct and four different sequence stretches of SmBMP were cloned into the Gal4-AD vector pACT2. The SmFst-containing plasmid was transformed into yeast cells (AH109) together with SmInAct or one of the BMP variants each. Following growth on selection plates (SD-Trp/-Leu/-His), β-gal liquid- and filter-assays were performed to relatively quantify interactions. These were shown between SmFst and SmInAct as well as between SmFst and SmBMP. The result of the β-gal liquid assay indicated a stronger binding of SmFst to SmInAct, while no evidence was obtained for an interaction with SmBMP-C-term or negative controls (Figure 7). This is surprising since this part of SmBMP is most conserved in comparison to human BMPs and contains several residues essential for receptor binding, which are supposedly blocked by FST [76], [84]–[87]. However, it is possible that within its quaternary structure FST binds to other residues, besides those at the C-terminus, thereby prohibiting the receptor binding of BMP. Together with the results from the localization experiments, evidence is provided that SmFst interacts with SmInAct and/or SmBMP in the testes of EM and UM but also in the female ovary, and in case of SmBMP in the vitellarium.

## Discussion

The results of our study provided conclusive evidence for pairing-influenced transcriptional processes in males. Together with previous findings about pairing-dependent gene transcription in females and first transcriptome analyses for males, all data clearly demonstrate a bidirectional transcriptional influence during male-female interaction [6], [7], [36], [37], [88], [89]. Our expectation to find a comprehensive data set of genes differentially transcribed in males upon pairing was met, just as obtaining congruent and complementing results using two independent techniques. Since both are basically different, each generated additional data that were not obtained by the other method. From our point of view, both methods have their advantages and disadvantages, and none seemed superior over the other. Both methods required the application of different statistics since on the one hand signal intensities were determined (microarray) including technical replicas and on the other hand transcript counts, both resulting in different log_2_-ratios. Finally, also biological variability, a well-known phenomenon even within schistosome strains used for such kind of analyses [54], may have had an influence on the data obtained. Although different methodological and analytical approaches were applied, an overlap of interesting genes was obtained on the basis of stringent analysis criteria. Taking into account that we made use of a second-generation microarray, which contained the majority of genes known from *S. mansoni* [42], [43], as well as SuperSAGE, which can theoretically detect all genes transcribed [47] the most complete data set of genes differentially transcribed between EM and UM was obtained. The credibility of the data was confirmed by the intersection of differentially transcribed genes identified by both techniques, but also by additional real-time PCR experiments. Furthermore, a comparison to data previously generated in other studies [36], [37], [90], [91] supported the reliability of our results. Waisberg et al. [90] found a number of genes for which transcription in males was influenced by final-host sex. Analyzing the transcription of the top 30 of these genes with our data sets revealed no significant differences between EM and UM. Thus an influence of the host sex within our experimental setup can be excluded. Of the transcripts differentially regulated between male stages or strongly up-regulated in at least one male stage found by Fitzpatrick et al. [37], we found 7 to be differentially regulated by SuperSAGE, including a Ftz-F1 interacting protein (Smp_090140.2), and 14 were differentially regulated according to microarray analysis, including several genes encoding metabolism-associated proteins. From the overlap of differentially transcribed genes between SuperSAGE and microarray only two genes, the AMP-dependent ligase and dock, were found within the data-set obtained by Fitzpatrick et al. [37]. Here, transcriptional regulation showed the same direction as in our study. Our data also confirmed results of one study previously applying SAGE to reveal differences between EM and UM [36]. As far as comparison was possible, a purin nucleoside phosphorylase (Smp_090520) was up-regulated in our microarray analysis as well as in the dataset of Williams et al. [36]. Also a cationic amino acid transporter (Smp_123010) (down-regulated in EM), a fatty acid binding protein (Smp_095360.x) (down-regulated in EM), and a heterogeneous nuclear ribonucleoprotein k (Smp_065580.x) (up-regulated in EM) showed the same direction of regulation in our study and were significant within our SuperSAGE data set.

Among the transcripts identified in our study to be regulated between EM and UM were also putative antisense RNAs. Some of these may have yet unknown protein-coding function, however, the majority of these RNAs are probably non-coding RNAs (ncRNAs). Their discovery in eukaryotic genomes has significantly influenced research recently, and first evidence for regulatory functions of ncRNAs has been obtained [91]. Also for *S. mansoni* the existence of antisense RNAs was reported [42], and it was estimated recently that ≥10% of the transcriptome may represent ncRNAs [43]. Life-stage analyses indicated alterations in the occurrence of specific ncRNAs pointing to diversified functions in biological processes [43], and we observed antisense RNAs (microarray: 211; SAGE-exon: 261; SAGE-intron: 107) that are differentially transcribed between EM and UM. Their analysis will be subject of future studies, when more knowledge about this class of molecules will be available for schistosomes. This applies also for the number of “hypothetical proteins” identified as being differentially transcribed (Supplementary Table S4), which could add up to the list of interesting candidates for further analyses of their potential function during pairing-associated processes in males.

Applying stringent analysis criteria and focusing on sense-transcripts only, a number of candidate genes were identified, which may be responsible for male competence and/or inducing female maturation. Data interpretation based on a combination of bioinformatics tools permitted first important conclusions (i–iv). According to GO-analyses (i) EM seem to loose complexity with regard to functional categories. This interpretation is supported by a previous study, in which less enriched GO categories were found in EM applying a first-generation microarray containing a lower number of gene-representing oligonucleotides [37]. In the same study the authors concluded that worms from mixed-sex populations are transcriptionally less complex than those from unisexual populations. Because in females paired to UM the induction of mitotic activity was found to be delayed compared to the situation in females paired to EM [29], it was hypothesized that EM and UM differ with respect to their mitosis-inducing capacity, what we like to define as competence that has to be reached before males have the full capacity to govern developmental processes in their pairing partners. The biological variety of transcripts belonging to distinct functional categories found to be differentially regulated indicated that (ii) gaining male competence is a process in which different systems are involved. This may also apply to male factors inducing female maturation. Although the involvement of neuronal processes [15] as well as sperm or seminal fluid [12], [14] as players during male-female interaction was dismissed in the past, (iii) we obtained first evidence that neuronal and testes-associated factors nevertheless may be involved. IPA analysis for genes of the intersection highlighted among others DDC, which was further accentuated through the metabolomic data-analysis with DDC as one of two molecules identified. Thus, together with the enhanced DDC transcript level in EM, our data suggest the possibility that neurotransmitters such as dopamine could play a role during male-female interaction. With regard to testes-associated genes, (72 genes were found in our study to be differentially transcribed according to microarray or SuperSAGE, which were previously detected as transcriptionally up-regulated in EM testes compared to whole worms [55]. These included dock and the OxlT (Smp_135020). Our *in situ*-hybridization experiments for two members of TGFβ-pathways as well as the OxlT (Smp_036470) (data not shown) localized transcripts for these molecules to the testicular lobes of EM and UM. The differential regulation of molecules like OxlTs, known for their participation in the indirect proton pump of *Oxalobacter formigenes* [92], (iv) may indicate their pairing-dependent function in metabolism processes. Previous studies already suggested that EM support females by supplementing their partner with nutrients [21]–[24], for which they might need a wider functional assembly of molecular processes than UM. In this context it is noteworthy that base metabolic processes seem to differ between the two male stages for anabolic and catabolic pathways, indicating higher nucleic acid synthesis rates in UM. Also, transcription of enzymes involved in carbohydrate metabolic processes, citrate cycle, aerobic respiration, and amino acids metabolic processes was rather up-regulated in UM. Metabolic differences between EM and UM were also found by Williams et al. [36].

The GCP protein was previously reported to be up-regulated in EM as a result of the male-female interaction [25], [27], [93] and proposed to be essential for pairing in *S. japonicum* [26]. However, transcriptional differences between EM and UM for GCP were neither confirmed by our data, nor by previous transcriptome analyses [36], [37], [88], [89]. This discrepancy could be explained by post transcriptional and/or post translational regulations. Interestingly, GCP was proposed to be a downstream target of TGFβ-pathways in schistosome couples [28]. This pathway is well known in schistosomes and was previously pointed out for its possible importance in the female reproductive biology being involved in regulating mitosis and egg production [9], [10]. Here we provide first evidence for an additional role of TGFβ-pathways during schistosome development as shown by the discovery of SmFst, a potential inhibitor of the TGFβ-pathway. Besides its down-regulation in EM, confirmed by all analyses, SmFst stood out in the IPA network identified for genes within the intersection of microarray and SuperSAGE data. Besides SmFst and its two potential interaction partners SmInAct and SmBMP, two other members of TGFβ-pathways were tested in real-time PCR experiments, Smad4 (Smp_033950) and one *S. mansoni* activin receptor (Smp_144390). Apart from SmFst none of the transcripts was differentially transcribed between EM and UM.

First functional studies demonstrated that SmFst transcripts were present in male testes and female reproductive organs. Furthermore, Y2H experiments confirmed its potential to interact with SmInAct and SmBMP. In *in situ*-hybridization experiments SmFst and SmInAct both localized in the female ovary, while SmFst and SmBMP each localized in male testes and the female reproductive organs. In addition, organ-specific RT-PCRs indicated the presence of all three transcripts in EM-testes and the ovary. A previously described transcriptome study [37] did not detect SmFst to be differentially regulated between EM and UM. This may be due to the absence of the corresponding oligonucleotide on the first-generation microarray used (the respective annotation was not found in the data-set [37]), which represented about 50% of the *S. mansoni* transcriptome. Within the *S. mansoni* organ-specific transcriptome data [71] SmFst was not found to be up-regulated in EM-testes compared to whole worms using a cutoff for 2-times higher transcription. Compared to our results, this seems not surprising since the EM transcript-level of SmFst was generally low, about two times weaker than in UM according to the calculated log_2_ratios.

With respect to transcript detection in the ovary, results of a previous report on SmInAct [82] corresponded to our localization data. While similar SmInAct transcript levels between EM and UM were detected in the earlier study and our analysis, SmInAct protein was only detected in EM and EF but not in UM or UF before, which suggested that SmInAct expression is linked to the reproductive capacity of the worm [82]. Assuming that SmInAct is the preferential binding partner of SmFst as shown in other organisms [78], [79], [94] two possible scenarios exist. First, transcript levels detected for SmFst in this study may not be representative for translation, and thus SmFst interactions would not occur in UM. Secondly, SmFst is translated and may influence TGFβ-pathways in the male testes, however, through other TGFβ-agonists such as SmBMP. Indeed, besides our Y2H results interactions between SmFst-SmBMP have been shown also in other organisms, where they are among others involved in gonad-specific developmental and physiological processes [64], [65], [95]–[100]. However, although the presence of SmBMP protein was demonstrated, it was not detected within the testes yet [83], which may have been caused by protein amounts below the detection limit.

From all results available today, we hypothesize a tissue- and stage-dependent interplay of TGFβ-family proteins in schistosomes that also affect the gonads. This view is supported by the presence of various type I and II receptors in the *S. mansoni* genome [30], [33]. Most type I receptors belong to the sub-group of activin-like receptors. Among these is an alternatively spliced variant of the *S. mansoni* TGFβRII [28], previously described as ActRII [98]. Other members are TGFβRI [100] (Smp_049760), ActRI (Smp_093540.3), ActRI/BMPRIa (Smp_124450), ActRIIa (Smp_080120.2) or ActRIIb (Smp_144390). Since multiple receptor-combinations are possible as well as their activation by promiscuously acting agonists [101], numerous interactions are imaginable with the potential to govern tissue-specific activities. In this scenario, SmFst may regulate TGFβ signaling by binding agonist such as SmBMP in testes of UM, which may impede processes not needed or not intended to occur before pairing. Whether the role of SmFst in schistosomes covers modulating agonist activities in the extracellular environment, or whether it is involved in processing SmInAct or SmBMP pre-pro-peptides to become pro-peptides, a normal part of the activation of these agonists [102], remains unclear at this stage of its analysis but will be subject of further studies. Although typical furin cleavage sites, being necessary for processing BMP or activin proteins in vertebrates [103], are present in the schistosome homologs, a role of SmFst in SmInAct or SmBMP protein processing appears unlikely since the main function of FSTs known today is its antagonist activity in the extracellular environment. Here it was shown that FSTs among others play prominent roles in the gonads controlling different testicular and ovarian functions including cell proliferation, apoptosis, folliculogenesis, luteogenesis, hormone release and fertility [104], [105].

In summary, the presented results demonstrate that processes leading to male competence may be far more complex than hypothesized before by reports suggesting that single molecules from the male and/or nutritional support could be in charge of the fundamental consequences of pairing on female development. From our results we conclude that besides metabolic processes, neuronal processes may be involved in the initial phase of male-female interaction but also TGFβ-signaling, which has been described before to be involved in differentiation processes in fully developed females [9], [10] and embryogenesis [82]. The meaning of this pathway for schistosome biology appears to go beyond that, since SmFst has leaped into view emerging as a regulatory molecule for TGFβ signal-transduction pathways that is pairing-dependently transcribed in the male gonad probably contributing to processes leading to male competence.

## Supporting Information

Figure S1 Depicts the molecular network suggested by IPA.
(TIF)

Figure S2 Depicts the sequence of SmFst, indicating differences between KC165687 and Smp_123300, as well as *in situ*-hybridization probes.
(TIF)

Figure S3 Shows stage-specific RT-PCRs for SmFst.
(TIF)

Figure S4 Gonad-specific RT-PCRs. Total RNA of isolated testes (Te), ovaries (Ov) and adult schistosome couples (SC) as control was isolated by Trizol and reverse transcribed [58]. RT-PCRs were performed using gene-specific primers targeting SmInAct, SmFst, and SmBMP. Marker (M) = Hyperladder II (Bioline).
(TIF)

Table S1 Shows results from the assignment of differentially regulated genes from either microarray or SuperSAGE to predicted *S. mansoni* metabolic pathways.
(XLSX)

Table S2 Lists members of the network generated by IPA, based on the genes found to be significantly differentially transcribed by both methods.
(TIF)

Table S3 Gives information on real-time PCR experiments. All primers used are listed, their concentration, annealing temperature and efficiency given. A second data-sheet shows the functional grouping of selected genes and summarizes results from microarray, SuperSAGE and real-time PCR.
(XLSX)

Table S4 Overview of individual data sets. **Sheet1**: 29 genes differentially transcribed between EM and UM according to both methods. log_2_ratios are given. **Sheet2**: Sense transcripts detected by the microarrays. Given are oligo_IDs, significances, log_2_ratios (highlighted by background coloring), Gene_IDs, annotations and detection for each stage in each microarray as 1/0. **Sheet 3**: Sense transcripts detected by SuperSAGE. Given are Gene_IDs, log_2_ratios, significances and transcript counts for each stage and experiment. **Sheet 4**: Genes differentially transcribed between EM and UM according to the microarrays only. Given are oligo_IDs, significances, log_2_ratios, Gene IDs, annotations and presence of *NlaIII* sites, as far as tested. Sheet 5: Genes differentially transcribed between EM and UM according to SuperSAGE only. Given are Gene_IDs, significances, log_2_ratios, according microarray probes and annotations.
(XLSX)
