# Supplementary figures and images for: Combinatory Microarray and SuperSAGE Analyses Identify Pairing-Dependently Transcribed Genes in Schistosoma mansoni Males, Including Follistatin
Source: PLoS Negl Trop Dis. 2013 Nov 7;7(11):e2532. doi: 10.1371/journal.pntd.0002532 (PMC3820750; doi:10.1371/journal.pntd.0002532)

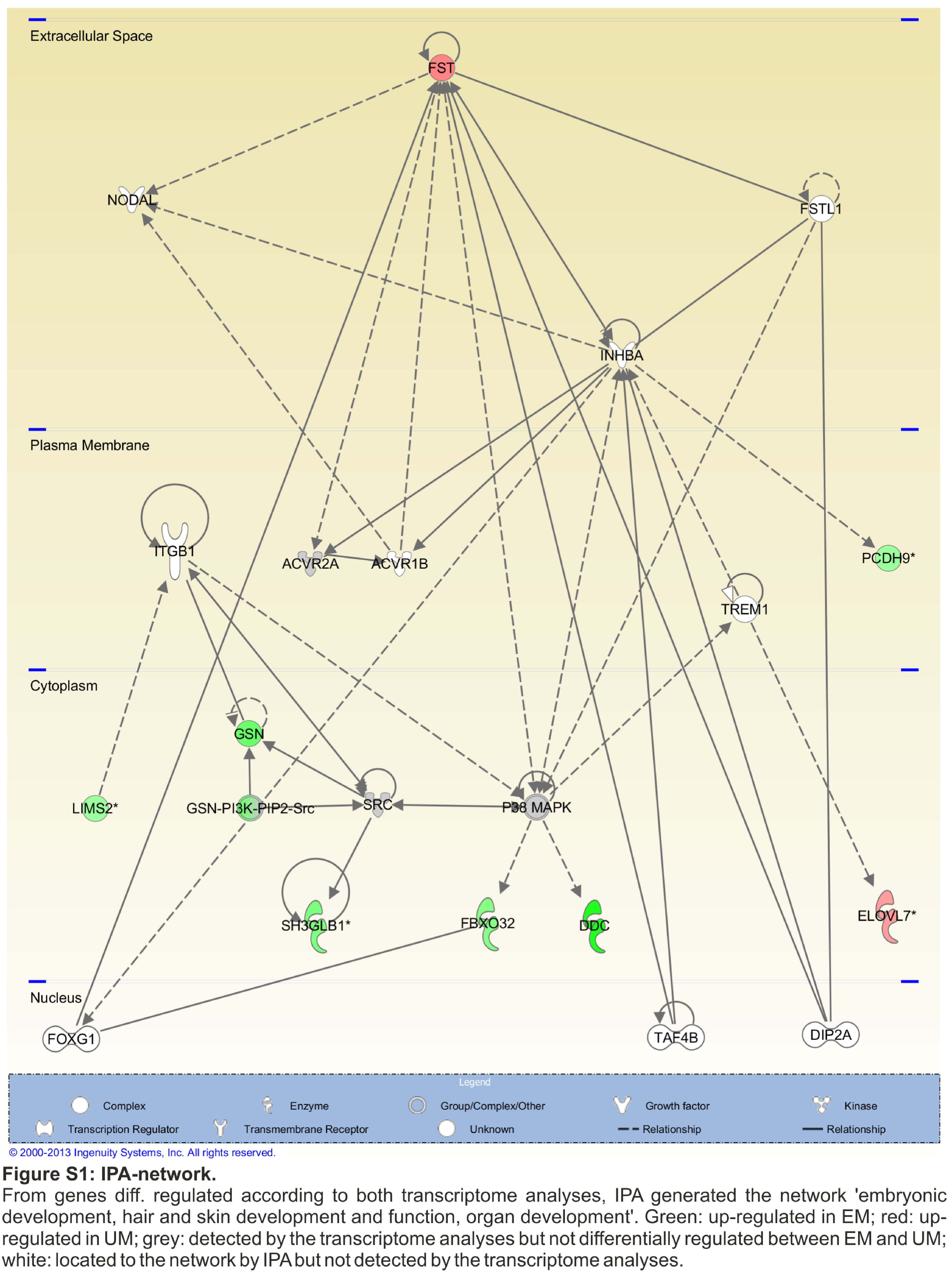

Supplement: Figure S1 — Depicts the molecular network suggested by IPA. (TIF) [file pntd.0002532.s001.tif]

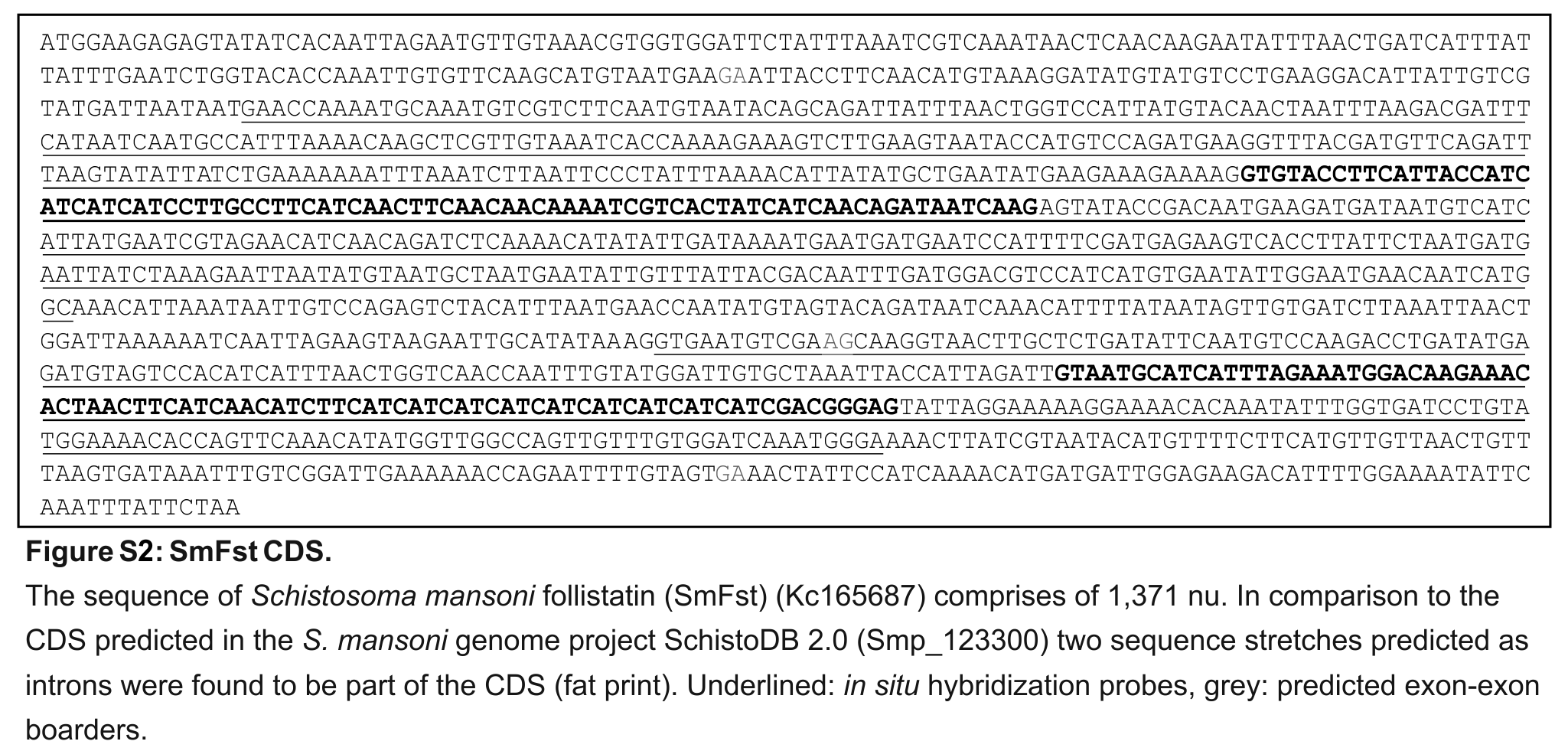

Supplement: Figure S2 — Depicts the sequence of SmFst, indicating differences between KC165687 and Smp_123300, as well as in situ-hybridization probes. (TIF) [file pntd.0002532.s002.tif]

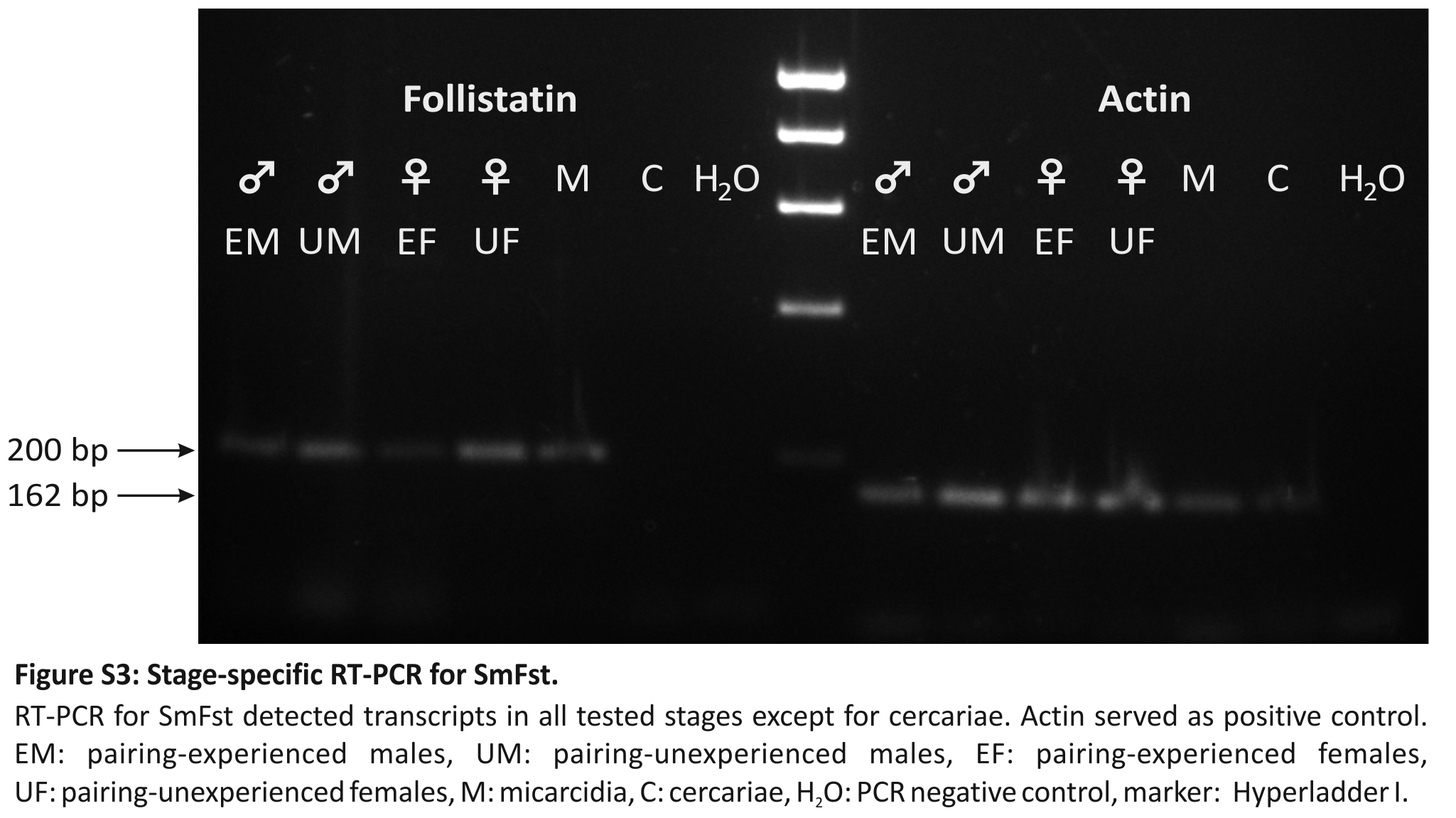

Supplement: Figure S3 — Shows stage-specific RT-PCRs for SmFst. (TIF) [file pntd.0002532.s003.tif]

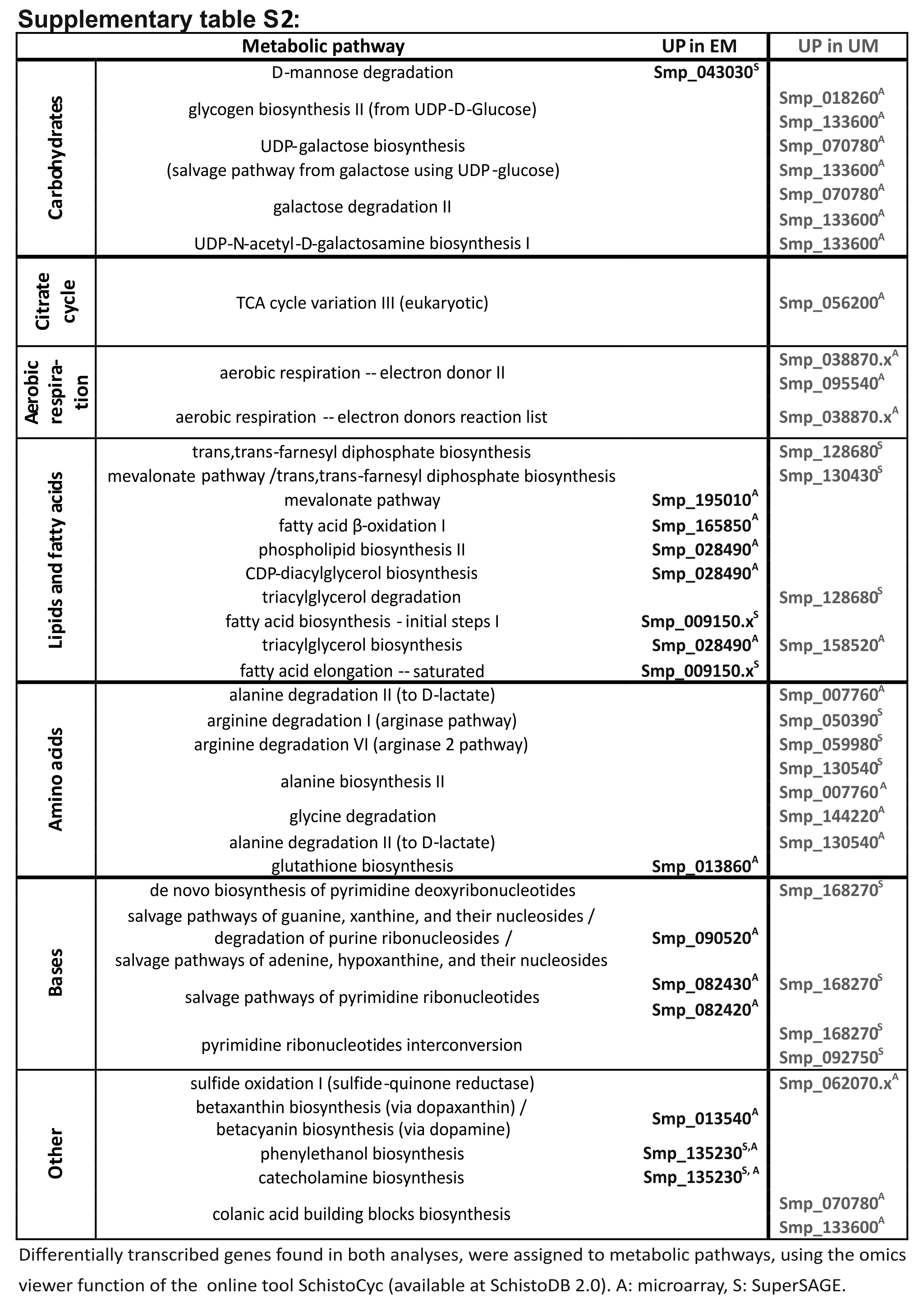

Supplement: Table S2 — Lists members of the network generated by IPA, based on the genes found to be significantly differentially transcribed by both methods. (TIF) [file pntd.0002532.s006.tif]
